# F145. WHAT ARE THE MAIN BRAIN CHANGES IN FMRI AFTER TREATMENT IN FIRST EPISODE PSYCHOSIS? A SYSTEMATIC REVIEW

**DOI:** 10.1093/schbul/sby017.676

**Published:** 2018-04-01

**Authors:** Carlos Gonzalez, Olga Sparano, Pau Soldevila-Matias, Gracian Garcia-Marti, Luis Marti-Bonmati, Benedicto Crespo-Facorro, Julio Sanjuan

**Affiliations:** 1 Clinic Hospital INCLIVA; 2 Ribera Hospital; 3 Quiron Hospital; 4 La Fe Hospital, Quiron Hospital; 5 Marques de Valdecilla Hospital, Santander; 6 University of Valencia

## Abstract

**Background:**

There are many studies using structural MRI to explore the longitudinal course of F Episode Psychosis (FEP).2 On the other hand, there is a lack of functional MRI studies examining the longitudinal course of FEP. The aim of this work is to make a literature systematic review of these studies, to summarize the knowledge about longitudinal course of functional brain activity in FEP.

**Methods:**

We followed the PRISMA guidelines for conducting systematic reviews and combined the use of electronic and manual systematic search methods, in the principal databases (MedLine, PubMed and Web of Science) using the query “longitudinal” AND “fMRI” AND “first episode psychosis” OR “first episode schizophrenia”. This search included (PERIODO). The inclusion criteria were: a) FEP diagnose; b) at least 2 functional MRI scans (pre-post); c) both task and resting-state scans were included. The exclusion criteria were: a) chronic patients in the studied sample; b) structural imaging results; c) just 1 fMRI scan; d) reviews and metaanalysis.

**Results:**

202 records were identified through database searching. A total of 10 articles were selected. From them, a total of 276 FES patients were examined by fMRI. In all of these studies patients were diagnosed by structured interviews according to DSM-IV-TR or ICD-10 criteria. The average age of the FES sample was 26.64 years old. Nine of the 10 studies involved 2 scans with a mean interval between them of 7 months. Six of the 10 studies did the first scan without any antipsychotic treatment, but all of them had medication at follow-up scan. Most of the studies used a region of interest (ROI) approach, and examined the role mainly of these areas: limbic system, hippocampus, striatum and prefrontal cortex.

Five of the studies used a resting-state paradigm. The other 5 works implemented some cognitive or emotional task using some visual stimuli.

Attending to the imaging results at baseline, most of studies found an hypoactivation of several brain areas, specially the limbic areas, like thalamus, amygdala and hippocampus. There are some other areas less activated compared to controls, including striatum, anterior cingulated cortex, orbitofrontal cortex, temporal gyrus and cerebellum posterior lobe.

At follow-up, almost all studies reported normalization of the hypoactivation levels found at baseline in the same regions. When the results at baseline were an increased activation, it also normalized at follow-up. There is only one study reporting an increased activity at baseline comparing to healthy volunteers which is still increased at follow-up scans.

**Discussion:**

There are very few studies exploring fMRI longitudinal course of FEP patients.

Our results in FEP are similar with other recent reviews in chronic schizophrenia samples,1 finding normalization (increase) of brain activity after antipsychotic treatment.

There are only visual or resting-state paradigms during scanning, which could explain some of the results. More investigations, involving other paradigms and related with psychopathological changes are needed, to test how the brain of the FEP patients change over time.

**References:**

1. Kani, A. S. (2017). Converging effects of diverse treatment modalities on frontal cortex in schizophrenia: A review of longitudinal functional magnetic resonance imaging studies. Journal of Psychiatric Research, 84, 256–276. J.JPSYCHIRES.2016.10.012

2. Gong, Q. (2016). A selective review of cerebral abnormalities in patients with first-episode schizophrenia before and after treatment. American Journal of Psychiatry, 173(3), 232–243.

